# Mosquito Surveillance Revealed Lagged Effects of Mosquito Abundance on Mosquito-Borne Disease Transmission: A Retrospective Study in Zhejiang, China

**DOI:** 10.1371/journal.pone.0112975

**Published:** 2014-11-13

**Authors:** Song Guo, Feng Ling, Juan Hou, Jinna Wang, Guiming Fu, Zhenyu Gong

**Affiliations:** Zhejiang Provincial Center for Disease Control and Prevention, Hangzhou, China; Institut Pasteur, France

## Abstract

Mosquito-borne diseases (MBDs) are still threats to public health in Zhejiang. In this study, the associations between the time-lagged mosquito capture data and MBDs incidence over five years were used to examine the potential effects of mosquito abundance on patterns of MBDs epidemiology in Zhejiang during 2008–2012. Light traps were used to collect adult mosquitoes at 11 cities. Correlation tests with and without time lag were performed to investigate the correlations between MBDs incidence rates and mosquito abundance by month. Selected MBDs consisted of Japanese encephalitis (JE), dengue fever (DF) and malaria. A Poisson regression analysis was performed by using a generalized estimating equations (GEE) approach, and the most parsimonious model was selected based on the quasi-likelihood based information criterion (QICu). We identified five mosquito species and the constituent ratio of *Culex pipiens pallens*, *Culex tritaeniorhynchus*, *Aedes albopictus*, *Anopheles sinensis* and *Armigeres subalbatus* was 66.73%, 21.47%, 6.72%, 2.83% and 2.25%, respectively. The correlation analysis without and with time lag showed that *Culex* mosquito abundance at a lag of 0 or 1 month was positively correlated with JE incidence during 2008–2012, *Ae. albopictus* abundance at a lag of 1 month was positively correlated with DF incidence in 2009, and *An. sinensis* abundance at a lag of 0–2 months was positively correlated with malaria incidence during 2008–2010. The Poisson regression analysis showed each 0.1 rise of monthly mosquito abundance corresponded to a positive increase of MBD cases for the period of 2008–2012. The rise of mosquito abundance with a lag of 0–2 months increased the risk of human MBDs infection in Zhejiang. Our study provides evidence that mosquito monitoring could be a useful early warning tool for the occurrence and transmission of MBDs.

## Introduction

Vectors such as mosquitoes are closely related with human health and mosquito-borne diseases (MBDs) have brought heavy social burdens in China. Approximately 30 million malaria cases occurred in China each year before 1949 in history, with some localities having parasite incidence rates as high as 80,000/100,000 [1]. The nearest outbreaks of dengue fever in Guangdong and Yunnan in 2013 have greatly influenced the local economic development and social stability [2]. With the increasing acceleration of global warming, large-scale urbanization and the rapid development of international tourism and trade, the density and distribution of mosquitoes may have undergone new changes [3]–[5]. Not only have some new MBDs emerged, but also the epidemic range and intensity of some reemerging MBDs have changed a lot [6], [7]. The MBDs such as dengue have already been the key issues of prevention and control of infectious diseases in southern China [8]. Therefore, mosquito surveillance, which can detect timely abnormal changes in population dynamics of mosquitoes, plays a very important role in early warning of the occurrence of MBDs and vector control strategies [9], [10].

Zhejiang province which has a typical subtropical climate lies in the southeast coast of China, and the topographic features in this province are complicated, including plains, hills and mountain lands, forming an ideal condition for the growth of mosquitoes. According to historical records and monitoring results for infectious diseases, there were imported and indigenous cases of Japanese encephalitis (JE), malaria and dengue fever (DF) diagnosed in this province. The primary vector of JE virus is *Cx. tritaeniorhynchus* and this virus was also isolated in *Cx. pipiens pallens* in Zhejiang [11], which could be regarded as the secondary vector for JE virus. The number of JE cases has been small because of planned child immunization, and the average annual incidence in this area was between 0.1/100,000 and 0.5/100,000 in the last 20 years [12], however, a large number of long-staying workers and their children who may not have JE vaccines from other provinces are the susceptible populations of JE that cannot be ignored [13]. *An. sinensis* is the only vector for malaria which has ever been a major health problem in Zhejiang, in October of 2011, Chinese government launched a plan to eliminate malaria in the country by 2020 [14]. Malaria incidence has been relatively low over the last several years in Zhejiang, and the average incidence rate in this area was 0.8/100,000 during 2004–2008 [15]. The first year that no indigenous patient was reported at Zhejiang was in 2012. Historical epidemics of dengue in Zhejiang had been documented in 1929, and no dengue outbreak was reported in the subsequent 76 years [16]. *Ae. albopictus* is the only vector for dengue virus transmission in Zhejiang. In 2004, a DF outbreak caused by an indigenous patient who had traveled from Thailand occurred in Cixi, a city in the northeast of Zhejiang, and 83 people were infected [17]. The largest epidemic occurred in 2009, and there were 196 cases in Yiwu [18].

Patterns of infection vary by time owing to extrinsic and intrinsic factors such as pathogens, host population, immunity, vectors, and climate [19], [20]. Incidence patterns reflect the complex interaction of all of these factors. The incidence of vector-borne diseases varies by year and month in Zhejiang, showing that potential changes in factors affect the transmission of diseases. Some studies [21]–[23] demonstrated a link between vector-borne diseases and climate change. Díaz et al. [24] reported that temperature, rainfall and sea-surface temperature were positively correlated with the number of dengue cases. Mosquitoes are the essential links in the transmission of MBDs, it is also widely accepted that the distribution and dynamics of MBDs cases are particularly sensitive to meteorological conditions, which largely depends on the sensitivity of the mosquitoes themselves and their blood-feeding patterns to variations in temperature, relative humidity and rainfall [25]–[27]. Therefore, the surveillance and control in mosquitoes are still the important part in routine work and emergency responses of MBDs.

In this study, we completed a 5-year monitoring work of mosquitoes, and analyzed the monitoring data of mosquitoes and MBDs at Zhejiang province during 2008–2012, in an effort to see the species, distribution and abundance change of mosquitoes at Zhejiang, and to qualitatively and quantitatively assess the relation between mosquito abundance and incidence of MBDs on a large scale to provide supports for MBDs early warning and control measures.

## Methods

### Ethics Statement

No specific permits were required for the described field studies: a) no specific permissions were required for these locations/activities; b) these locations are not privately-owned or protected; c) the field studies did not involve endangered or protected species. Ethical approval was not required because the study was conducted as part of surveillance control management for national notifiable diseases. Incidences of MBDs in different cities were calculated for analysis, and there was no specific information of patients used in this study. Patient records/information was anonymized and de-identified prior to analysis.

### Mosquito capture data

**Figure 1:**
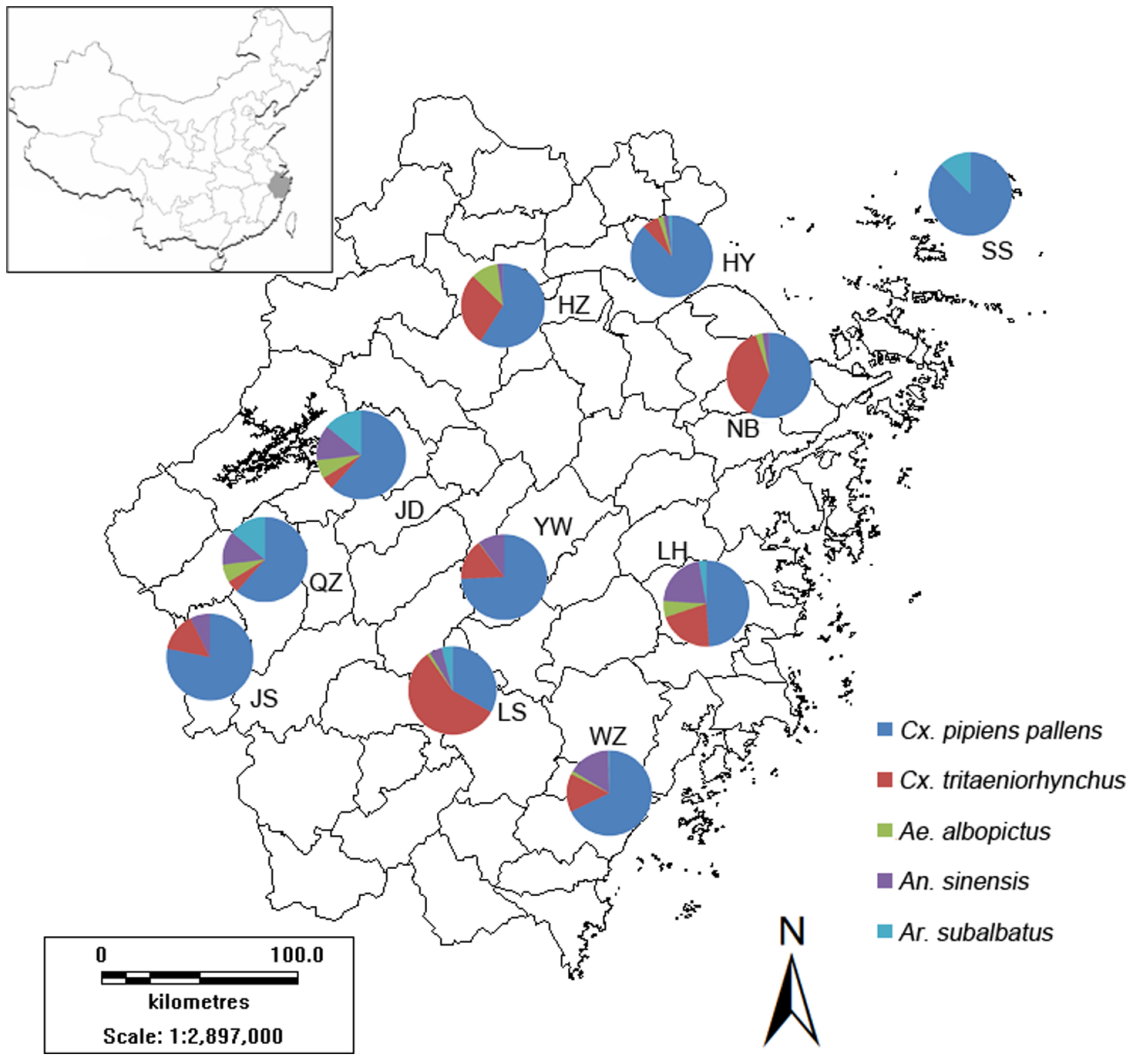
Monitoring cities and mosquito species in Zhejiang. The map shows 11 monitoring cities and mosquito species in each city. Pie charts display the proportion of mosquito species in each monitoring city during 2008–2012. Dark blue represents *Cx. pipiens pallens*, red represents *Cx. tritaeniorhynchus*, green represents *Ae. albopictus*, purple represents *An. sinensis*, and sky blue represents *Ar. subalbatus*.

According to different geographical features and epidemic history of MBDs, there were 11 monitoring cities chosen (Figure 1, Table 1): Wenzhou, Quzhou, Lishui, Jiande, Haiyan, Shengsi, Jiangshan, Linhai, Yiwu, Hangzhou and Ningbo. Kung Fu Xiaoshuai miniature light traps [28] (Photocatalytic Miewen Ying supply device; Wavelength: 2537 Å; Power: 8 W; Corporation: Wuhan Environmental Protection Technology Co., Ltd. Gemstar) recommended by Chinese center for disease control and prevention (CCDC) were used to collect adult mosquitoes. Five mosquito species (*Culex pipiens pallens*, *Culex tritaeniorhynchus*, *Aedes albopictus*, *Anopheles sinensis* and *Armigeres subalbatus*) closely associated with human beings were identified. Traps were hung away from interference by light sources, 1.5 m above the floor. Every monitoring city consisted of two urban residential communities, two public parks, two public hospitals, two rural residential areas and two live-stock sheds with one light trap placed in nonprivately-owned areas of each site. We chose one day in the middle of a month for monitoring, and the monitoring time was from 18 o 'clock to next morning. The research was conducted from April to November. Adult mosquito abundance is defined as the average number of mosquitoes collected per trap night.

**Table 1:** Details for 11 monitoring cities in the study.

Regions	Monitoring cities	Code	Geographical coordinates
Eastern coast	Haiyan	HY	30.53°N, 120.95°E
Eastern coast	Ningbo	NB	29.88°N, 121.56°E
Eastern coast	Linhai	LH	28.86°N, 121.15°E
Eastern coast	Wenzhou	WZ	28.00°N, 120.69°E
Eastern coast	Shengsi	SS	30.72°N, 122.47°E
Central part	Hangzhou	HZ	30.29°N, 120.15°E
Central part	Yiwu	YW	29.32°N, 120.07°E
Central part	Lishui	LS	28.45°N, 119.92°E
Western part	Jiande	JD	29.48°N, 119.29°E
Western part	Quzhou	QZ	28.95°N, 118.88°E
Western part	Jiangshan	JS	28.74°N, 118.63°E

### Disease data

We collected cases for JE, DF and malaria from National Notifiable Disease Report System (NNDRS) in Zhejiang. All cases were diagnosed according to the unified diagnostic criteria issued by Chinese Ministry of Health (MOH). JE should be considered in a patient with evidence of a neurologic infection (e.g., meningitis, encephalitis, or acute flaccid paralysis) in Zhejiang. Laboratory diagnosis of JE is generally accomplished by testing of serum or cerebrospinal fluid (CSF) to detect virus-specific IgM antibodies. In fatal cases, nucleic acid amplification or virus culture of autopsy tissues would be used to diagnose. Dengue can be diagnosed by isolation of the virus, by serological tests, or by molecular methods (one-step, real time RT–PCR or nested RT–PCR). Malaria should be considered in a person with symptoms (most often fever, chills, sweats, headaches, muscle pains, nausea and vomiting) and physical findings (elevated temperature, perspiration, tiredness). Microscopic diagnosis and other specialized tests such as serology and PCR would be used in confirmed diagnosis.

### Statistical methods

We carried out a correlation analysis without and with time lag to investigate the lagged effects with a lag of 0 to 2 months of the mosquito abundance on monthly MBDs incidence rates through observation of statistical significance from the period of 2008–2012. The correlations between JE and *Culex* mosquitoes (including *Cx. pipiens pallens* and *Cx. tritaeniorhynchus*) were calculated for JE virus that was isolated from two above-mentioned species of mosquitoes in Zhejiang. DF was assessed with its only vector *Ae. albopictus* and malaria was assessed with its only vector *An. sinensis*. Spearman rank correlation coefficient *r_S_* or Pearson product-moment correlation coefficient *r_P_* was calculated depending on the data type (non-Gaussian or Gaussian distributed).

The lagged-time Poisson regression analyses were performed by using SAS Version 9.3 for Windows (SAS Institute Inc., Cary, North Carolina, USA). A basic univariate Poisson regression model can be written as,
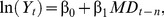
(1)where *Y_t_* is the incidence of confirmed cases at time *t*, β_0_ is the intercept, β_1_ represents coefficients, *MD is* the monthly mosquito abundance, and *t−n* in the subscript represents the *n*-month lag time.

The monthly disease incidence was modeled using a generalized estimating equations (GEE) approach with a Poisson distribution. The most parsimonious model was selected based on the quasi-likelihood based information criterion (QICu) [29], [30]. To quantify the effects of mosquito abundance, we computed the influences (e^(0.1*β)^−1), which correspond to the percent increase.

## Results

### Mosquito species and distribution

**Figure 2:**
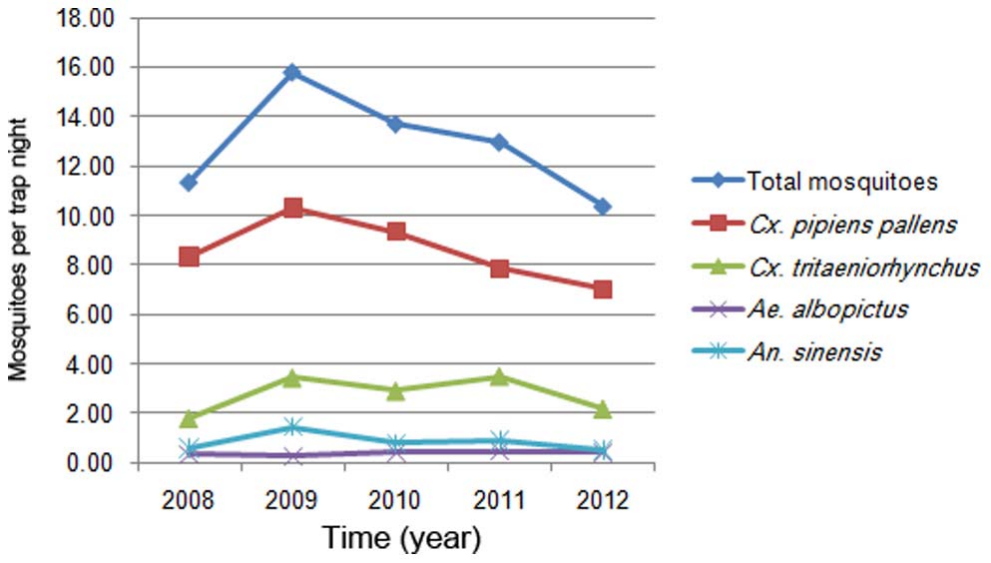
Annual abundance of five mosquito species based on 11 cities during 2008–2012. Dark blue line represents abundance of total mosquitoes, red line represents abundance of *Cx. pipiens pallens*, green line represents abundance of *Cx. tritaeniorhynchus*, purple line represents abundance of *Ae. albopictus*, and sky blue line represents abundance of *An. sinensis*.

The number of identified five mosquito species was 89060 from 2008 to 2012, and included *Cx. pipiens pallens*, *Cx. tritaeniorhynchus*, *An. sinensis*, *Ae. albopictus* and *Ar. subalbatus* (Figure 1, Table 2). Mosquitoes of up to 90 percent were *Cx. pipiens pallens* and *Cx. tritaeniorhynchus*. The constituent ratio of *Cx. pipiens pallens*, *Cx. tritaeniorhynchus*, *An. sinensis*, *Ae. albopictus* and *Ar. subalbatus* was 66.73%, 21.47%, 6.72%, 2.83% and 2.25%, respectively. The catches of mosquitoes by light traps were quite different among all monitoring cities. Haiyan, a city in the north of Zhejiang, had the highest catches, while Yiwu which lies in the center of this province had the fewest mosquitoes captured. Additionally, differences in mosquito species existed among some monitoring cities, we did not find any *Cx. tritaeniorhynchus* or *An. sinensis* at Shengsi, there was no *Ae. albopictus* found at Jiangshan and no *Ar. subalbatus* found at Hangzhou, Ningbo, Quzhou, Yiwu and Jiangshan either. The changes of mosquito abundance by year based on 11 cities are shown in Figure 2. The shape of abundance change of *Cx. tritaeniorhynchus* displayed an M type curve, which was the same as the curve of the total mosquito abundance, while the shape of abundance change of *Cx. pipiens pallens* presented unimodal curves, the peak value of annual *Cx. pipiens pallens* abundance occurred in 2009.

**Table 2:** Mosquito catches in each monitoring city during 2008–2012.

	HY	HZ	NB	WZ	LH	JD	QZ	YW	JS	LS	SS	Total	Constitution rates (%)
*Cx. pipiens pallens*	14022	4413	8529	10241	2154	2336	3100	1901	4471	3298	4961	59426	66.73
*Cx. tritaeniorhynchus*	1045	2122	5625	2201	917	178	125	393	826	5688	0	19120	21.47
*Ae. albopictus*	346	781	384	191	267	257	177	5	0	111	3	2522	2.83
*An. sinensis*	322	162	383	2413	932	489	147	256	423	461	0	5988	6.72
*Ar. subalbatus*	200	0	0	54	125	533	0	0	0	400	692	2004	2.25
Total	15935	7478	14921	15100	4395	3793	3549	2555	5720	9958	5656	89060	

### Correlation analysis

**Figure 3:**
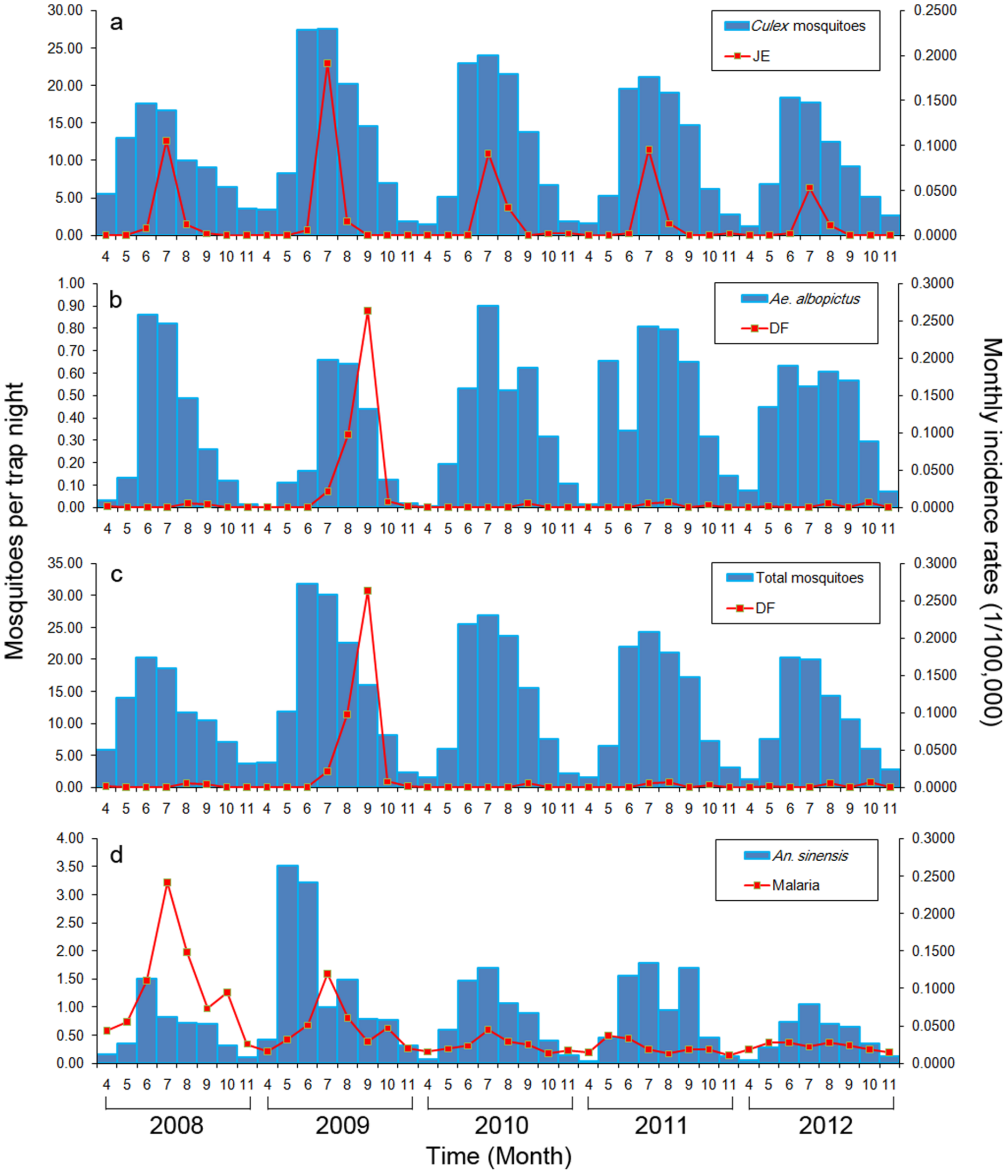
Monthly mosquito abundance and MBDs incidence rates. (a) *Culex* mosquito abundance and JE incidence rates; (b) *Ae. albopictus* abundance and DF incidence rates; (c) total mosquito abundance and DF incidence rates; (d) *An. sinensis* abundance and malaria incidence rates.

The mosquito abundance changes and incidence of MBDs by month are shown in Figure 3, selected MBDs consist of JE, DF and malaria. The shapes of mosquito abundance changes by month were represented as unimodal or bimodal type curves. There were noticeable lagged effects between monthly mosquito abundance and monthly incidence of MBDs, and peak values of mosquito abundance and MBDs incidence occurred in the same month or delayed by one or two months (Figure 3, Table 3). For JE and *Culex* mosquito abundance, they showed statistically significant correlation at 0-month lag in 2009, 2011 and 2012 (2009: *r_S_* = 0.846, *p*<0.01; 2011: *r_S_* = 0.723, *p*<0.05; 2012: *r_S_* = 0.791, *p*<0.05) performed by the Spearman's correlation analysis, and there were positive correlations between JE incidence and mosquito abundance in 2008 and 2010 at a lag of one month (2008: *r_S_* = 0.939, *p*<0.001; 2010: *r_S_* = 0.753, *p*<0.05). For DF and *Ae. albopictus* abundance, just the local dengue outbreak caused by imported cases in 2009 was analyzed. The peak value of dengue incidence was two months behind the peak value of *Ae. albopictus* abundance, and there was positive correlation between them at a lag of one month (*r_S_* = 0.927, *p*<0.001) performed by the Spearman's correlation analysis. For malaria and *An. sinensis* abundance, there were positive correlations between them at 0-month lag in 2010 (*r_P_* = 0.838, *p*<0.01), at a lag of one month in 2008 (*r_P_* = 0.910, *p*<0.01) and at a lag of two months in 2009 (*r_P_* = 0.831, *p*<0.05) performed by Pearson's correlation analysis.

**Table 3:** Results of correlation coefficient for time-lag effects.

Time-lag (months)	Mosquito abundance				
	2008	2009	2010	2011	2012
(A)					
0	0.685	**0.846** **	0.434	**0.723** *	**0.791** *
1	**0.939** ***	0.682	**0.753** *	0.583	0.709*
2	0.545	0.000	0.230	−0.203	0.027
(B)					
0		0.708*			
1		**0.927** ***			
2		0.563			
(C)					
0	0.531	0.061	**0.838** **	0.221	
1	**0.910** **	0.698	0.739*	−0.367	
2	0.211	**0.831** *	0.247	−0.568	

The correlation coefficients are calculated between (A) monthly JE incidence rates and time-lagged *Culex* mosquito abundance, (B) monthly DF incidence rates and time-lagged *Ae. albopictus* abundance, (C) monthly malaria incidence rates and time-lagged *An. sinensis* abundance, respectively. Boldface denotes the largest value of correlation coefficient and significance with

*p<0.05;

**p<0.01; and

***p<0.001.

All significance levels are assessed at α<0.05.

### Poisson regression analysis

Based on the correlation analysis with time lag, we chose the lagged mosquito abundance with MBDs incidence in every separate year for Poisson regression analysis. Table 4 lists the best-fitting models with the smallest QICu values to characterize the relationships between monthly cases and mosquito abundance. Mosquito abundance at lags had positive effects on MBDs incidence. Each 0.1 rise of monthly *Culex* mosquito abundance corresponded to an increase of 3.01% (95%CI 1.11% to 4.95%) to 9.25% (95%CI 0.16% to 19.17%) in the monthly number of JE cases for the period of 2008–2012, each 0.1 rise of monthly *Ae. albopictus* abundance corresponded to an increase in the monthly number of DF cases by 7.81% (95%CI 3.47% to 12.34%) in 2009, and each 0.1 rise of monthly *An. sinensis* abundance corresponded to an increase of 0.49% (95%CI 0.23% to 0.74%) to 1.30% (95%CI 0.84% to 1.76%) in the monthly number of malaria cases during 2008–2010.

**Table 4:** Statistics of best-fitting Poisson regression models of the monthly cases (2008–2012) on the mosquito abundance.

	β	S.E.	*p*	QICu	(e^(0.1*β)^−1) = percent increase (%)	95% CI for percent increase (%)	
						Lower boundary	Upper boundary
(A)							
2008(Lag1)	0.6886	0.2092	<0.01	−84.9509	7.13	2.83	11.61
2009(Lag0)	0.3164	0.0803	<0.0001	−37.7425	3.21	1.60	4.85
2010(Lag1)	0.2968	0.0951	<0.01	−23.9126	3.01	1.11	4.95
2011(Lag0)	0.8847	0.4433	<0.05	3.9918	9.25	0.16	19.17
2012(Lag1)	0.5577	0.2780	<0.05	−15.9544	5.74	0.13	11.66
(B)							
2009(Lag1)	7.5208	2.0986	<0.001	−70.6459	7.81	3.47	12.34
(C)							
2008(Lag1)	1.2891	0.2314	<0.0001	−241.0632	1.30	0.84	1.76
2009(Lag2)	0.4842	0.1293	<0.001	−124.6235	0.49	0.23	0.74
2010(Lag0)	0.5830	0.1278	<0.0001	−575.0240	0.58	0.33	0.84

The Poisson regressions are calculated between (A) monthly JE cases and time-lagged *Culex* mosquito abundance, (B) monthly DF cases and time-lagged *Ae. albopictus* abundance, (C) monthly malaria cases and time-lagged *An. sinensis* abundance, respectively. All significance levels are assessed at α<0.05.

## Discussion

Five mosquito species identified as vectors of important infectious diseases were well established in Zhejiang province. The incidence of MBDs in the province have been lower than the average level in China for the last several years [31]–[33], however, the prevention and control of MBDs have been challenged by the existence of travel-related cases and the abundance of long-staying migrant workers [34]. The presence and patterns of abundance have been obtained for mosquito species in different regions and this provides baseline data for relating to presence of associated vector-borne diseases. While no *Cx. tritaeniorhynchus* were found on the islands of Shengsi, this needs continuous monitoring for the potential invasive species because of the potential for establishment with the constant urbanization process and intensive island exploitation activities. Vector surveillance is critical for early warning and control strategies of disease, and an integrated MBDs surveillance should consist of aspects of people, vectors, pathogens, reservoir hosts and climate [35]–[37], so the study of the relationship between MBDs and influencing factors is an effective supplement for case-based surveillance system.

The lagged effects were apparent in the relationship between mosquito abundance and MBDs incidence, and the lagged time was no more than two months in most years, as well as the peak value of monthly MBDs incidence and monthly mosquito abundance. This was consistent with the incubation periods of MBDs. The incubation period for JE varies from 5 to 15 days [38], for DF, the incubation period is from 3 to 14 days [39], and the incubation period in most cases varies from 9 to 40 days for malaria [40]. Mosquito abundance could serve as an effective indicator for the increase of MBDs cases. It is considered that climatic factors such as rainfall and temperature affect the occurrence of vector-borne diseases, essentially, the climatic factors change the disease incidence through affecting life cycles of mosquitoes or changing in susceptibility to some pathogens [41], [42], Lebl et al. [43] found that they have predicted mosquito abundances by interval lagged weather data with a feasible accuracy, especially when related to weekly *Cx. pipiens/restuans* populations. However, some studies have demonstrated that human activities and their impact on local ecology have generally been much more significant, while climate has rarely been the principal determinant of disease's prevalence or range [44]. The relationship between vector-borne diseases and climatic factors is much more complicated than it appears. Data on mosquito abundance are more convenient to detect short-term possible outbreak and develop mosquito control strategies. The lagged time of one or two months is fundamental for prediction and vector management in communicable disease control, it may not prevent the occurrence of MBDs outbreaks, and nevertheless, we can shorten the period of outbreaks and reduce the number of cases at the pandemic peak.

There is a positive quantitative relation between monthly mosquito abundance and monthly cases. These quantitative relations can be used as theoretical basis for vector-borne diseases prevention and control. The increase of *Culex* mosquito abundance was coincident with a distinct rise of JE cases, though high vaccine immunization rates in populations greatly reduce the incidence of JE, the mosquito control still cannot be ignored. There is not targeted vaccine for malaria, but the preventive measures and therapy for malaria are mature and effective, the percent increase of malaria cases was low than other MBDs with the increase of *An. sinensis* abundance. DF is the only vector-borne disease caused only by imported cases in Zhejiang, there is no specific treatment for dengue/severe dengue, dengue prevention and control solely depends on effective vector control measures. The increase of DF cases was little higher than other MBDs with the increase of *Ae. albopictus* abundance. However, there was an underestimate on *Ae. albopictus* abundance through the method of light traps as adult *Ae. albopictus* mosquitoes prefer daytime activity, the actual percent increase may even larger than this calculated value. The *Ae. albopictus* abundances over the five years were approximate, but the peak value of total mosquito abundance in 2009 was the highest during the five years (Figure 3c), which also indicated that there may be an underestimate on *Ae. albopictus* abundance in 2009. Mosquito abundance still gave evidence for potential dengue outbreaks.

The impact of time-lag effect on MBDs incidence found in this study had also been supported by some studies. Barrera et al. [45] found that peaks in mosquito abundance preceded maximum dengue incidence and oviposition was significantly correlated with dengue incidence in San Juan, Puerto Rico. Tadei et al. [46] reported that a relationship between *An. darlingi* abundance increases and the number of malaria cases 30 days later was observed in Manaus. Barros et al. [47] found the decrease in distribution and abundance of anophelines corresponded to a decrease of malaria incidence in surrounding areas. Martins-Campos et al. [48] also indicated there was strong evidence of association between the abundance of *An. darlingi* and the incidence of malaria.

Some limitations must be acknowledged. First of all, owing to this investigation being an ecological study, although we emphasized the impact of mosquito abundance, we could not exclude other potential factors. Secondly, we chose to use monthly aggregated data of diseases and monthly average or aggregate mosquito data, the results would be more accurate if weekly data were used. We chose monitoring frequency for once a month mainly because the mosquito abundance was similar during a month in Zhejiang from historical records, and the MBDs incidence has been low for the last several years. Thirdly, there was only one year data to analyze the association between *Ae. albopictus* and the outbreak of dengue fever, for Zhejiang is not the natural epidemic focus for DF virus and the number of dengue outbreak is limited. The association between *Ae. albopictus* and the outbreak of dengue fever needs more evidences to be proved.

In conclusion, we reported that a rise of mosquito abundance with a lag of 0–2 months increased the risk of human MBDs infection in Zhejiang. There are positive quantitative relations between monthly mosquito abundance and monthly cases. Our study provided evidence that mosquito monitoring could be a useful early warning tool for the occurrence and transmission of MBDs, and vector surveillance is an effective supplement for case-based surveillance system of MBDs. MBDs may not threaten to shut down the economy of Zhejiang, yet it's a growing killer that deserves some attention of its own.
